# A Tool to Guide Creation of Products for Risk Communications and Community Engagement (RCCE)

**DOI:** 10.3389/fpubh.2022.810929

**Published:** 2022-05-27

**Authors:** Wai Jia Tam, Nina Gobat, Divya Hemavathi, Dale Fisher

**Affiliations:** ^1^Yong Loo Lin School of Medicine, National University of Singapore, Singapore, Singapore; ^2^Nuffield Department of Primary Care Health Sciences, University of Oxford, Oxford, United Kingdom; ^3^Infectious Diseases Division, University Medicine Cluster, National University Hospital, Singapore, Singapore

**Keywords:** Risk Communication and Community Engagement (RCCE), COVID-19, vaccination, vaccine hesitancy, creation tool

## Abstract

Using best practices to produce creative, relatable, contextualized health messaging contributes to effective risk communication. During emergency disasters, the landscape of mis- and dis-information demands strategic, collaborative approaches across all stakeholders particularly government and the media to ensure effective public messaging. However, tools for new RCCE practitioners and media agencies such as television producers and advertising firms to rapidly create effective RCCE products are currently not readily available. In response to concerns that vaccine hesitancy may become more evident once a significant proportion of the population had been reached, the Ministry of Communications and Information (MCI) in Singapore launched a public health music video on 2 May 2021, making headlines globally and garnering more than 5 million views worldwide. The video aimed to dispel myths and concerns about vaccinations and encouraged citizens to get their vaccination quickly rather than wait. We aimed to evaluate this video as a case study and articulate why it is an example of good practice in risk communications. Working inductively to identify emergent principles of product creation in this case study and analyzing them against existing RCCE frameworks and recommendations helped develop a practical tool to guide the rapid creation of RCCE products by those who may be unfamiliar to RCCE principles. This tool can help new RCCE practitioners and media agencies to produce effective products in times of crisis. The easy-to-use tool provides a brief checklist that guides rapid creation of RCCE products, including criteria for understanding the target audience, message comprehension, development, reach and impact measurement. Given its derivation from existing RCCE frameworks and health literacy concepts, these can potentially be applied across different modalities and diverse cultures. Future work would include validation of these criteria and evaluation of its utility to strengthen RCCE as core in an emergency response.

## Introduction

Risk communication, an integral part of any emergency response, is the real-time exchange of information, advice and opinions between experts, community leaders, or officials and the people who are at risk of disease (1). It aims to influence behaviors, attitudes and social norms in order to promote adherence with public health and social measures that can slow the spread of infection and protect health. To achieve these aims, effective risk communication must capture the public's attention, permeate the right communication channels, convey accurate messages in a timely manner, and be accessible across languages, ages, cultural affiliations and education levels (2). During health crises such as the COVID-19 pandemic, the landscape of mis- and dis-information demanded strategic, collaborative approaches across governmental, media and other agencies to ensure effective public messaging. Nonetheless, tools for new RCCE practitioners and media agencies such as television producers and advertising firms to rapidly create effective RCCE products are currently not readily available (2). Effective risk communication practices draw from entertainment, education and communication theories. As such, in a disaster, Risk Communication and Community Engagement (RCCE) practitioners often work closely with media agencies such as television producers and advertising agencies who may not be well-versed with RCCE principles. While tools that evaluate the effectiveness of RCCE messages, as well as recommendations and frameworks that guide RCCE strategy exist, practical tools such as checklists to create specific RCCE products like video advertisements within a short turnaround are currently not readily available for those new to RCCE (1, 3–5). This tool intends to provide quick and relevant guidance for new RCCE practitioners and advertisers or media producers working closely with RCCE agencies for maximal success. Due to time constraints in an emergency, this easy-to-use, brief checklist aims to be highly applicable and easily implementable even for those unfamiliar with RCCE.

## Context

This paper presents a case study of a vaccination promotion video produced in Singapore to promote uptake of COVID-19 vaccines. Singapore's COVID-19 vaccination programme began on 30 December 2020, targeting healthcare and frontline workers (6). This was extended to those in the community aged 70 years old and above on 27 January 2021, followed by those aged 45 years and above on 24 March 2021 (6, 7). As of 9 May, about 1.8 million individuals (32% of the total population) had received at least one dose of the vaccine, and of which, about 1.2 million (21%) completed a 2-dose course (8). A survey in Singapore found that about 50% of the population would want to receive the COVID-19 vaccine the moment it is available. However, as many as 34% would wait six to 12 months, 9% would consider having it eventually, and the remaining 9% said they would not choose to be vaccinated at all (9). Particularly, individuals aged 36 years old and above exhibited high levels of vaccine hesitancy (10). Reasons for vaccine hesitancy included the fast track development of the vaccine, concerns about its efficacy and adverse side effects, the risk averse mentality of senior citizens, concerns about existing chronic disease and low numbers of community cases contributing to a low risk perception of COVID-19 as a health threat in Singapore (11). These views were suspected to be more prevalent among the older age groups.

In response to growing concerns regarding vaccine hesitancy, especially among those 45 years old and above, the Ministry of Communications and Information launched a public health music video on 2 May 2021 through the digital government portal (www.gov.sg) and shared it on social media channels, making headlines globally and garnering more than 5 million views worldwide. The video aimed to dispel myths and concerns about vaccinations and encouraged citizens, especially those 45 years and older, to have their vaccination quickly rather than wait.

In Singapore, the population comprises a majority of Chinese ethnicity (77%), followed by a minority of Malays (15%), Indians (7%) and other races (1%) (12). Languages spoken include English (37%), Mandarin (35%), Chinese dialects (12%), Malay (11%), Tamil (3%), and other languages (2%) (13). Studies have also shown that about 60% of individuals aged above 40 prefer to obtain information from online sources (14). As an example of good practice in risk communications, we sought to evaluate the vaccination promotion video technically since it had been well-received locally and globally, with the aim of developing an easy-to-use tool to guide rapid creation of effective RCCE products (3). In our literature review, we identified RCCE frameworks, health literacy concepts for risk communication, and evaluation tools (4, 5, 9, 11). However, there was no easily available tool to guide the rapid creation of RCCE products such as this video (1, 15–18). We identified this as a gap as during emergency disasters, the landscape of mis- and dis-information demands strategic, collaborative approaches across governmental, media and non-profit agencies to ensure effective public messaging and new RCCE practitioners and media agencies such as television producers and advertising firms who are unfamiliar with RCCE principles need guidance for such specific communications.

## Key Programmatic Elements

### Development of Foundational Frameworks and Concepts

Relevant concepts and frameworks for RCCE such as the WHO guidelines for risk communication, health literacy concepts and RCCE best practices were identified through a literature review on best practices in the entertainment and communication industry and evaluation tools in RCCE (1, 4, 5, 9, 11).

### Applying These Concepts to the Video

We transcribed and analyzed the video for content and health messages and reviewed the images too. The style and delivery were also considered against the concepts and frameworks, and a checklist was created by the authors. An in-depth semi-structured interview with the government agency that developed the video was conducted to uncover the processes involved in development. (See Appendix in Supplementary Table 1 for list of questions) Metrics measuring the outcomes of the video and public health impact were sought (1, 2). Responses were analyzed inductively adopting thematic analysis. Emergent principles and best practice guidelines from the interview were distilled. Findings were evaluated against concepts and frameworks for risk communication, and a simple checklist for creation of RCCE products was developed (1, 4, 5, 9, 11). The in-depth interview revealed that the video was commissioned by Ministry of Communications and Information (MCI), partnering closely with Ministry of Health. The video was part of a larger integrated nationwide vaccination campaign entitled “I Got My Shot.” Targeted at Singaporean residents aged 45 years and above, who were likely consumers of the heartland comedy sitcom *Phua Chu Kang*, the music video was launched alongside a series of videos in three main vernacular languages; Chinese, Tamil and Malay, to achieve the needed exposure (19).

The music video, entitled “Vaccinated Chu Kang,” was conceptualized in March 2021 and launched in early May during the vaccination rollout among those aged 45 years and above. It adopted a light-hearted tonality, familiar celebrity characters as well as everyday Singaporeans to ensure it was relatable. Scenes shot in the vernacular videos portrayed ordinary citizens of specific ethnicities practicing *taichi* at community centers, shopping for groceries at markets, and having tea at coffeeshops, to depict vaccination as an important everyday conversation to have with one's friends and family. The videos were shared on television, supported by the campaign's print advertisements on bus stands, newspapers and in public housing estates. The line-by-line analysis of the video revealed 3 main messages: Do not hesitate in getting vaccinated, vaccination is safe for those with chronic diseases and mild allergies, and vaccination is beneficial for the community.

### Creating the Tool Based on Practice-Based Experience

The WHO's most updated risk communication guidelines describe three ambitions; building trust and engaging with affected populations, integrating emergency risk communication into health and emergency response systems and practicing emergency risk communication as a continuous process (1). Combining these recommendations with best practice guidelines in health literacy, entertainment and communication industries, and insights from the in-depth interview with the video development team, our team established 5 key factors important for RCCE product development including (see items 5.1 to 5.5 below).

In addition, the authors created a table to serve as an easy-to-use tool for future RCCE product development efforts aimed particularly at new RCCE practitioners and media agencies, in response to emergencies for effective health messaging (See Supplementary Table 2) (1, 3, 11, 15–17, 20–28).

## Findings

Research through analysis of and interaction with the target community should commence once the RCCE objective is established. Often in a disaster, a detailed audience profile cannot be thoroughly undertaken but considerations include the nature of the audience, whom they trust, what they believe and what concerns motivate their actions. Risk communication will suffer to the extent that the audience is mischaracterized (29).

### Understanding the Target Audience

In our case study, a hired research vendor conducted surveys online and phone interviews with at least 1,000 Singaporean residents aged 15 years and above about their sentiments on vaccination. This data was triangulated with qualitative on-ground feedback obtained by Silver Generation ambassadors (senior citizen health ambassadors deployed by Ministry of Health to do regular house visits) and multi-agency feedback units (30, 31).

Findings revealed that people aged 45 years and above were concerned about the efficacy of the vaccine, and that chronic disease and existing allergies might affect their ability to tolerate the vaccine well, resulting in a wait and watch attitude.

Interviews with the Public Communications Division of Ministry of Communications and Information (MCI) revealed their focus on knowing their target audience well, including their preferred media platforms and vernacular languages used. Past research showed this age group preferred simple, humourous everyday language that was relatable and without technical jargon. As such, the emergent video was easy to understand, catchy and made use of a popular celebrity.

#### Use of Popular Celebrity Characters

The pro-vaccination video is played by popular comedian Gurmit Singh who starred in Phua Chu Kang Pte Ltd, a drama sitcom which ran from 1997 to 2007 on local television (19). Singh's popularity is proven by multiple awards and the success of a public service announcement rap made during the SARS crisis, humorously entitled “SAR-vivor!”(32). Evidence supports the use of celebrities as their endorsement can signal credibility and catalyse herd behavior, since cognitive dissonance is experienced when they do not (29). While Singh and the cast act as characters with eccentric personalities, it is possible that the influence of celebrity status has a part to play in diffusing through social networks and people's desire to acquire celebrities' social capital (29).

#### Local Contextualization and Authentication

Authenticity describes important technical content embedded in a context relatable to the target audience. Its importance is increasingly recognized in public health, with its longstanding challenges of audiences translating knowledge into action (29). While authenticity is rarely a feature in technical information in risk communication, it is an appraisal made by people persuaded to view the information as especially relevant to their health behaviors and consonant with their prior experiences. Public health communicators can encourage such appraisals by using narrative formats that provide rich contextualization and acknowledging a dialogic dimension to persuasion that aids in the process of authentication (29).

The music video is a combination of these crucial components. The use of characters from a long-running, familiar drama sitcom lends a relatable and distinctly Singaporean narrative format with rich contextualization. It also adopts a unique language known as “Singlish,” which is the country's distinct creole of English, Chinese dialects, Malay and Tamil, thus appealing directly to local audiences (29). References to humorous lines from the sitcom such as “Don't play play!” and Singaporean slangs like “Steady Pom Pi Pi” bridge the gap between authority-levels and community at large. Interestingly, in spite of its local contextualization, it remains understandable to diverse global viewers (2).

The team developed a checklist tool to ensure adequate understanding of the target audience including whether the target audience profile was known in terms of age, education level, their language and dialects, main sources of information, education and entertainment, their beliefs and concerns about the topic, habits, hobbies and which celebrities they admire.

### Message Comprehension

Health messages utilizing relatable narratives are more effective in changing behaviors than didactic recommendations of bland technical information (2, 25). While authorities note that laypeople do not understand highly technical risk information, communities perceive experts to be unable to understand their concerns (29). Often, there is a failure to recognize that individual biases and limitations may lead to distorted and inaccurate perceptions of risk problems that cannot be resolved by traditional, unilateral approaches (29).

The development team behind the video thus wrote the lyrics to the music video, ensuring short sentences written in local slang. The short duration of under 2 min of the video was intentional as was the repetitive chorus, allowing people to remember the messages easily.

Comparisons have been made between this video and those in New Zealand and Australia. Similar to Singapore, New Zealand adopted a humourous approach with local vernacular, declaring “Ka kite, COVID,” meaning “see you, COVID” in the Maori language. Likewise, there is a strong relatable narrative framing vaccination as “the pathway to freedom.” Both videos target different age and ethnic groups positively, reinforcing a utilitarian worldview that “vaccination is for the community not just the individual.” This approach was in contrast to Australia's, which adopted videos with a more somber tone by senior government advisors and specialist health professionals. We were unable to assess the videos from Australia and New Zealand against our checklist due to lack of access to the producers.

The campaign video of May 2021 in our case study built on the earlier March 2020 video with feedback that enabled the team to affirm the importance of message comprehension through the use of catchy tunes and simple lyrics to address ground concerns while ensuring message recall. Dialects and vernacular languages were applied more to improve for the later version.

Piloting the health messaging with a focus group of the target audience to gather feedback and ensure the message resonates with them adds to the likelihood of a successful product.

### Message Content and Development

A challenge to good risk communication is the provision of accurate, standardized, coordinated and timely messaging that is responsive to on-ground needs and sentiments. RCCE must be conveyed in a timely, accurate manner in order to establish trust and achieve its goals to reduce transmission (18). This often requires government, agency or organizational infrastructure to strategize, coordinate, build capacity and eventually scale (1).

#### Responsiveness to the Specific Context

In a previous music video in March 2020 at the start of the COVID-19 crisis, the same celebrity character uses a personal hygiene song to takes jabs at recent episodes of nationwide hoarding and panic buying, with rows of toilet paper and instant noodles appearing in the background, items which Singaporeans stockpiled (29). The relevance in the timeliness of the video was crucial to resonate with the public, building up early rapport and trust through this character, possibly contributing to the greater success achieved by the subsequent video.

#### Accuracy of Messages

The video contains several factually accurate and detailed health messages to build trust with the public. Accuracy of the message and its currency was ensured by collaboration with the Ministry of Health and pilot testing it with feedback groups. Understanding real time concerns via sensing mechanisms and having operational procedures for regular and nimble creation of technically accurate products is a crucial part of successful sustained community engagement.

#### Coordination of Production and Dissemination

The fact that the Ministry of Communications and Information (MCI) was dedicated to producing health messaging campaigns and coordinating their production and dissemination across various government agencies and ministries reflects the government's priority to provide RCCE response as a designated strategic role in the COVID-19 outbreak response.

The tool includes whether health messages are responsive to the current context, if they are factual and relevant, and whether there is a system of leadership infrastructure and governance in place to respond to evolving ground needs nimbly and to scale.

### Message Reach

The digital media landscape has changed the way information can be disseminated to populations (30). Increasingly, a variety of actors, including non-official sources, use social media to disseminate public service announcement (PSA)-like content (31). Good risk communication requires a capacity for a sustained two-way exchange so that ground concerns and feedback can be continually addressed and used to improve future messaging (19). This is an advantage of social media platforms, since digital audiences tend to be large, and have the ability to interact with content through shares, likes, or comments (31). The use of social media as a dissemination channel can also help counter misinformation, conspiracy theories, bots, and trolls (31).

Besides traditional media like radio and television, the video was also released on the government's social media platforms and website, Gov.sg. A multimodal dissemination strategy can reach people of varying literary and educational backgrounds. People with lower health literacy are more likely to trust television, social media, blogs, celebrity webpages, and friends, making this video easily accessible by all (33). The portrayal of government as being credible yet fun and friendly also helps build trust. Channels utilized by younger age groups were also targets for dissemination, to enable their sharing of videos with parents and grandparents.

The checklist in the tool developed by the team to ensure broad message reach includes media consumption platforms and practices of the target audience, and the extent of diversity in dissemination channels.

### Impact Measurement

Monitoring and evaluation indicators are important to measure impact, facilitate ongoing improvements and ensure sustainable funding. The tool suggests reviewing metrics and feedback on a regular basis.

Post-campaign online and phone surveys assessed the campaign's impact on the consumers' attitude, message recall and campaign effectiveness in nudging behavior and changing mindsets. The “Vaccinated Chu Kang” video registered a high awareness at 78% among the respondents. Performance metrics set by the team included number of views, length of time that the video was watched by viewers, qualitative comments received on social media and media commentary.

The willingness of respondents to take the COVID-19 vaccine when offered to them increased from 69% prior to the launch of the campaign in early April to 81% after the launch in mid-June. More than half of respondents who were initially undecided or chosen against vaccination indeed changed their minds and felt the campaign materials greatly or moderately helped in their decision to get vaccinated. Vaccination rates overall in Singapore displayed nearly 30% increase in the same time period (29).

Sole attribution cannot be given to the music video as many other efforts were taking place in parallel (5).

## Conceptual and Methodological Constraints

The tool to guide creation of RCCE products has limitations. Evaluation of the effectiveness of the single product will always be a challenge as they are never produced in isolation. For this reason, the tool focuses on creating the best opportunity for a good product. Views of representative members of target audiences as well as relevant stakeholder groups could provide useful qualitative feedback (3, 34, 35). Furthermore, the reliability and translatability of this tool is yet to be validated.

Establishing RCCE products during a crisis includes resource challenges including the availability of key personnel being available to create the product quickly. Regular feedback mechanisms should be leveraged on to understand community and multistakeholder sentiments, for the tool to be adequately conceived, produced and distributed. RCCE competes with other core pillars of outbreak response and is often inappropriately deprioritised.

While social media metrics are a proxy for dissemination and reach in the community, they do not reveal direct impact on behavioral change. It is also difficult to ascertain if the video produced had any significant direct impact on the target group. It is possible that the popularity of the video among the public could trickle on to the target group in the form of younger caregivers and family members urging target group members to take up the message instead of directly reaching the target audience. Focus group discussions among the target audience and their children who received the videos would more accurately reveal their sentiments toward the video and impact on perspectives. Challenges are also faced in directly correlating and attributing vaccination rates with social media metrics.

## Lessons Learnt and Future Direction

RCCE products are often created intuitively in emergency settings, during which, the landscape of mis- and dis-information demands strategic, collaborative approaches across governmental, media and non-profit agencies to ensure effective public messaging. While RCCE frameworks, concepts, recommendations and evaluation tools exist, we propose a simple tool aimed at new RCCE practitioners and media agencies such as television producers and advertising firms to rapidly create effective RCCE products, which is currently not readily available. Many such operators may not even appreciate how RCCE in an emergency differs from conventional advertising. The development of a tool can enable all collaborators to produce effective products in times of crisis. Future work would include validation of these criteria and evaluation of its utility to strengthen RCCE product creation in emergency settings.

## Data Availability Statement

The raw data supporting the conclusions of this article will be made available by the authors, without undue reservation.

## Author Contributions

WT, DF, and NG conceptualized the manuscript. DH contributed to the data collection and literature review. WT did the initial draft while DF, NG, and DH critically revised the manuscript. All authors have approved the final version.

## Conflict of Interest

The authors declare that the research was conducted in the absence of any commercial or financial relationships that could be construed as a potential conflict of interest.

## Publisher's Note

All claims expressed in this article are solely those of the authors and do not necessarily represent those of their affiliated organizations, or those of the publisher, the editors and the reviewers. Any product that may be evaluated in this article, or claim that may be made by its manufacturer, is not guaranteed or endorsed by the publisher.
