# Supplementary material for: A Tool to Guide Creation of Products for Risk Communications and Community Engagement (RCCE)
Source: Front Public Health. 2022 May 27;10:810929. doi: 10.3389/fpubh.2022.810929 (PMC9197119; doi:10.3389/fpubh.2022.810929)
Supplement: Supplementary file 1 [file Table_1.DOCX]

Appendix

Table 1: Questions asked at the in-depth interview with the video development team

| Who commissioned this work? |
| --- |
| When did it start being developed? |
| Who advised on the creation, development, and production of the work? |
| What was the kind of research that went into it? (e.g. surveys done, focus group discussions, people interviewed, research based on past successes of similar PCK videos etc) |
| In particular, what kind of research went into the target audience to ensure relatability? |
| What was the intended impact, and were there ways you measured the outcome/impact of the video on vaccination rates?  What are some metrics you used to measure the success of the video? |
| How much cost went into the production of this? |
| How long did it take? |
| What kinds of dissemination channels did you use to share the video? |
| How did your agency boost engagement/reach? |
| Is there anything you would have done differently? |
| Were there unexpected outcomes from the video? |
