# Supplementary material for: A Tool to Guide Creation of Products for Risk Communications and Community Engagement (RCCE)
Source: Front Public Health. 2022 May 27;10:810929. doi: 10.3389/fpubh.2022.810929 (PMC9197119; doi:10.3389/fpubh.2022.810929)
Supplement: Supplementary file 2 [file Table_2.docx]

Table 2: Tool for guiding creation of RCCE products

| Criteria for evaluation | | | Checklist |
| --- | --- | --- | --- |
| 1. Understanding of target audience | - Demographics - Language - Sources of information and entertainment - Beliefs about topic (e.g. vaccination) - Concerns about topic (e.g. chronic disease) - Habits (e.g. places they frequent, hobbies etc.) - Relatable celebrities - Local contextualization - Preferred tone (e.g. serious/formal, informal/personal, friendly) | |  |
| 1. Message comprehension | - Use of simple language - Use of local vernacular - Easy takeaways - Simple call-to-actions - Use of catchy tunes - Good health literacy- easy for audiences to access, view and understand health information | |  |
| 1. Message content and development | - Responsive to context - Factual and relevant messages - Infrastructure in place to respond to ground needs - Engage dedicated government agencies for emergency preparedness - Cross-organizational networks available for coordination - Training programmes available to build capacity for emergency responses - Budget for RCCE messaging | |  |
| 1. Message reach | - Use of traditional media platforms - Use of social media platforms | |  |
| 1. Impact measurement | - Social media metrics collated regularly (e.g. reach, engagement) - Traditional feedback conducted regularly (e.g. phone interviews, focus group discussions) | |  |
